# Impact of body image on women’s fitness persistence: the chain mediation role of self-efficacy and exercise motivation

**DOI:** 10.3389/fpsyg.2026.1737243

**Published:** 2026-04-01

**Authors:** Junhua An, Pengfei Tai, Jiahui Peng, Deqiao Zhou

**Affiliations:** 1 College of Physical Education and Health, Guangxi Normal University, Guilin, China; 2 School of Physical Education and Sport Science, Qufu Normal University, Jining, China; 3 School of Physical Education, Shandong University, Jinan, China

**Keywords:** body image, chain mediation, exercise adherence, exercise motivation, self-efficacy, women’s health

## Abstract

**Background:**

Body image, as a crucial cognitive-emotional factor in women’s fitness behavior, has garnered attention for its multifaceted influence on exercise persistence. However, the chain mediation it consconsently that involving self-efficacy and exercise motivation has not been fully elucidated.

**Objective:**

This study aims to explore the pathways through which body image is associated with women’s fitness persistence, and to examine the individual and chain mediation effects of self-efficacy and exercise motivation.

**Methods:**

A cross-sectional survey design was employed, involving 721 women aged 18 to 38 from Shandong Province. The instruments used included the Body Image State Scale, Exercise Persistence Scale, General Self-Efficacy Scale, and Exercise Motivation Scale. Data were analyzed using SPSS 27.0 and PROCESS 4.0, with correlation analysis, regression analysis, and bootstrap mediation effect testing.

**Results:**

Body image significantly and positively predicted fitness persistence (*β* = 0.695, *p* < 0.001), with 54.1% of the total effect explained through mediation pathways. The single-step mediation effect of self-efficacy was the strongest (22.8%), the chain mediation (body image → self-efficacy → exercise motivation → behavior) accounted for 19.4%, and the independent mediation by exercise motivation accounted for 11.9%. Notably, self-efficacy had the highest explanatory power (*R*^2^ = 46.7%), underscoring its pivotal role in sustaining behavior.

**Conclusion:**

Body image is related to long-term fitness persistence among women by enhancing self-efficacy and exercise motivation, particularly via their chain mediation mechanism. These findings provide theoretical support for intervention strategies centered on cognitive restructuring and motivational enhancement.

## 1 Introduction

Women’s fitness persistence is a pivotal topic in public health and psychology. Sustained engagement in physical activity not only reduces the risk of chronic disease but also supports mental well-being, making this line of inquiry central to population health promotion strategies (Bin and Yanwen, 2022). Within this context, body image—a core component of self-cognition—offers a theoretically relevant lens for understanding fitness persistence. Body image is related to how individuals evaluate their exercise-related capabilities and influences their motivation, thereby positioning it as an important antecedent of sustained exercise participation (Dian’e et al., 2022).

Among Chinese women, body image is increasingly shaped by the intersection of traditional aesthetic expectations emphasizing “femininity” and contemporary idealized body standards amplified by social media, which may generate distinctive tensions in body-related self-evaluation. At the same time, national initiatives emphasizing health in China have heightened women’s awareness of fitness; however, low persistence remains common (Sahin and Sanlier, 2025). Examining how body image relates to fitness persistence in this population therefore contributes to cross-cultural research in exercise psychology and may inform more targeted approaches to improving women’s health outcomes. In particular, positive body image is associated with persistence by strengthening self-identity and commitment to exercise, whereas body image anxiety driven by unrealistic beauty ideals may operate as a barrier to sustained participation (Tylka and Wood-Barcalow, 2015).

Accumulating evidence suggests that long-term adherence to fitness behaviors reflects not only physiological demands but also psychological and cognitive processes. For example, a mixed-methods study of women’s gym experiences reported that negative body image—expressed as perceived appearance- and performance-based judgments from others—elicited persistent feelings of inadequacy, which in turn undermined exercise self-efficacy and reduced willingness to continue exercising (Cowley and Schneider, 2025). Conversely, positive body image has been linked to higher exercise self-efficacy and, consequently, stronger adherence. Supporting this association, a cross-sectional survey of 1,102 college students showed that body image significantly mediated the positive relationship between physical activity and exercise self-efficacy (*β* = 0.383, *p* < 0.001) (Zhou et al., 2025). Nonetheless, prior work has predominantly emphasized direct associations between body image and fitness behaviors, and fewer studies have examined the internal pathways through which body image may translate into persistence—particularly an integrated cognitive–motivational chain mechanism.

Guided by social cognitive theory, self-efficacy is likely to serve as a key mechanism linking body image to fitness persistence: positive body image may strengthen women’s confidence in achieving fitness goals, whereas negative body image may erode beliefs about overcoming exercise-related barriers (Hessler et al., 2019). In parallel, self-determination theory highlights exercise motivation, especially internalized forms, as a central predictor of sustained behavior. Importantly, the directionality between body image and fitness persistence has not been fully resolved, as existing evidence suggests a bidirectional relationship: positive body image may facilitate continued exercise, while sustained exercise may also foster more positive body image through mastery experiences and perceived physical improvements. Given that body image is malleable rather than fixed, the cross-sectional design of the present study precludes causal inference and captures only concurrent associations, without establishing the temporal ordering required for causal conclusions. To strengthen interpretability, we adopt a bidirectional conceptual framework when discussing mediation results. We also recommend that future research employ longitudinal designs (e.g., 6–12 months of follow-up) or cross-lagged panel models to clarify reciprocal relations among body image, self-efficacy, and exercise behavior in women.

Against this background, the present study focuses on urban Chinese women who engage in fitness activities and examines the mechanisms through which body image is associated with fitness persistence, with particular attention to the sequential (chain) mediating roles of self-efficacy and exercise motivation. The study aims to contribute theoretically by (1) moving beyond single-mediator models to articulate a dynamic explanatory pathway—body-related cognition → efficacy beliefs → motivational regulation → behavioral persistence—and (2) situating women’s fitness psychology within the intertwined influences of China’s “slimness culture” and the emerging discourse of “healthy beauty” from a cross-cultural perspective. Practically, the findings may inform intervention design, such as addressing body image distortions through cognitive-behavioral approaches and enhancing persistence through stepwise goal setting to strengthen self-efficacy and internalized motivation.

## 2 Literature review and research hypotheses

### 2.1 Body image and women’s fitness persistence behavior

Existing evidence suggests that body image, as an important cognitive–affective determinant of women’s exercise behavior, is positively associated with fitness persistence through multiple pathways. Participation in regular resistance training or group exercise, for example, has been linked to improvements in body satisfaction. In addition, women with more positive baseline body image appear more likely to reframe their exercise goals from appearance compensation to functional enhancement, which may reduce dropout risk (Campbell and Hausenblas, 2009). One plausible explanation is an “achievement–recognition” cycle, whereby perceived progress reinforces positive body-related evaluations and strengthens continued engagement. Consistent with this account, Homan and Tylka’s work with college students indicated that body-appreciation–oriented motivation was associated with a 25% reduction in social comparison, which shifted attention toward personal performance improvement and was related to longer training duration (Homan and Tylka, 2014).

Longitudinal findings further highlight the sustained relevance of body image. In a six-month study of 328 women, body-related pride enhanced self-efficacy and was associated with a 31% increase in weekly exercise duration, whereas groups reporting high body shame exhibited dropout rates as high as 42% (Larson et al., 2019). From a behavioral ecology perspective, Raggatt et al. (2018) reported that women who followed bloggers promoting “functional beauty” increased exercise frequency by 1.5 times, while those oriented toward “ideal body standards” showed a higher risk of anxiety.

Cultural values may also shape the strength of these associations. Cross-group analyses suggest that in Western cultural contexts that emphasize bodily autonomy, body image explains a larger proportion of variance in exercise motivation than in East Asian samples, although the direction of association appears consistent across groups (Tylka and Homan, 2015). Collectively, this evidence indicates that body image may be understood as of body-related cognition that supports women’s fitness persistence by dampening negative affective feedback and fostering intrinsically oriented goals. Accordingly, we propose the following hypothesis:

*H1*. Body image is positively associated with women’s fitness persistence; women with more positive body image are more likely to persist in fitness activities.

### 2.2 The mediating role of self-efficacy

The emotional dimension of body image may shape how individuals attend to and interpret efficacy-relevant information. Tylka and Wood-Barcalow (2015) reported that individuals with higher body trust experience fewer attentional disruptions stemming from body-related anxiety in challenging situations, which may allow cognitive resources to be allocated more efficiently toward building self-efficacy (Anson et al., 2015). Related evidence from performance contexts suggests that a fluent sense of bodily movement can reinforce beliefs about one’s capacity to complete tasks in accordance with intended goals—for example, among dancers, embodied fluency has been linked to stronger confidence in task execution (Rhodes et al., 2016).

Beyond affective processes, the functional cognitive–behavioral dimension of body image may strengthen efficacy-related feedback through a behavioral “closed-loop” mechanism. Sabiston et al. path analysis indicated that exercise participation shaped by body image influences whether individuals accumulate mastery experiences, which in turn represent one of the most robust predictors of self-efficacy (Murphy et al., 2019). Converging empirical findings support these links: Gao et al. (2024) reported that body image significantly and positively predicts self-efficacy, and self-efficacy, in turn, is positively associated with exercise persistence. Taken together, these findings suggest that self-efficacy may serve as a key mechanism through which body image relates to women’s fitness persistence. Accordingly, we propose the following hypotheses:

*H2*. Body image is positively associated with self-efficacy.

*H3*. Self-efficacy is positively associated with women’s fitness persistence.

*H4*. Self-efficacy may mediate the association between body image and women’s fitness persistence.

### 2.3 The mediating role of exercise motivation

A growing body of research documents a positive association between body image and exercise motivation, an association that can be interpreted within social cognitive theory and self-determination theory (Leung et al., 2023; Navas-León et al., 2025). For instance, research among overweight and obese college students showed that body image predicted exercise persistence through exercise self-regulation, suggesting that more positive body image may strengthen individuals’ capacity for autonomous regulation of exercise behavior. Evidence from women’s samples further supports this pattern. Using body shame, body appreciation, and BMI to identify four body image profiles (BIPs), one study found that the Appreciative BIP was associated with the highest weekly exercise volume, whereas the High Shame BIP was associated with the lowest, underscoring positive body image as an important correlate of women’s fitness participation and persistence (Raspovic et al., 2023).

Beyond these direct links, prior evidence also points to a sequential (chain) mechanism in which self-efficacy and exercise motivation jointly transmit the influence of body image on fitness persistence. Specifically, positive body image may enhance exercise self-efficacy, which in turn promotes more autonomous forms of motivation and supports sustained participation; conversely, negative body image (e.g., body shame) may undermine exercise confidence, weaken motivational regulation, and increase the likelihood of dropout (Ouyang et al., 2020). Notably, gender differences have been reported: one cross-sectional study observed a stronger body image–persistence association among women than men, and body dissatisfaction was negatively related to women’s exercise persistence (Styk et al., 2024).

From the perspective of self-determination theory, body image may influence women’s exercise motivation by shaping the satisfaction of basic psychological needs (autonomy, competence, and relatedness), thereby facilitating the internalization of motivation. Exercise motivation—particularly intrinsic and other autonomous forms—has been consistently linked to greater persistence in exercise behavior. Based on this theoretical rationale and empirical evidence, we propose the following hypotheses:

*H5*. Body image is positively associated with exercise motivation.

*H6*. Exercise motivation is positively associated with women’s fitness persistence.

*H7*. Exercise motivation may mediate the association between body image and women’s fitness persistence.

### 2.4 The chain mediation role of self-efficacy and exercise motivation

Self-efficacy, a central construct in social cognitive theory, has been consistently linked to exercise motivation. Individuals with higher self-efficacy tend to set more challenging exercise goals and sustain participation by regulating effort and adopting effective coping strategies. Evidence from clinical contexts further supports this relationship: among individuals with chronic diseases, self-efficacy–enhancing interventions have been shown to increase exercise participation, and these gains are positively associated with the duration of maintained motivation (Klompstra et al., 2021). Building on this evidence and the preceding theoretical rationale, we propose the following hypotheses:

*H8*. Self-efficacy is positively associated with exercise motivation.

*H9*. Self-efficacy and exercise motivation sequentially mediate the association between body image and women’s fitness persistence.

### 2.5 Research model

**Figure 1:**
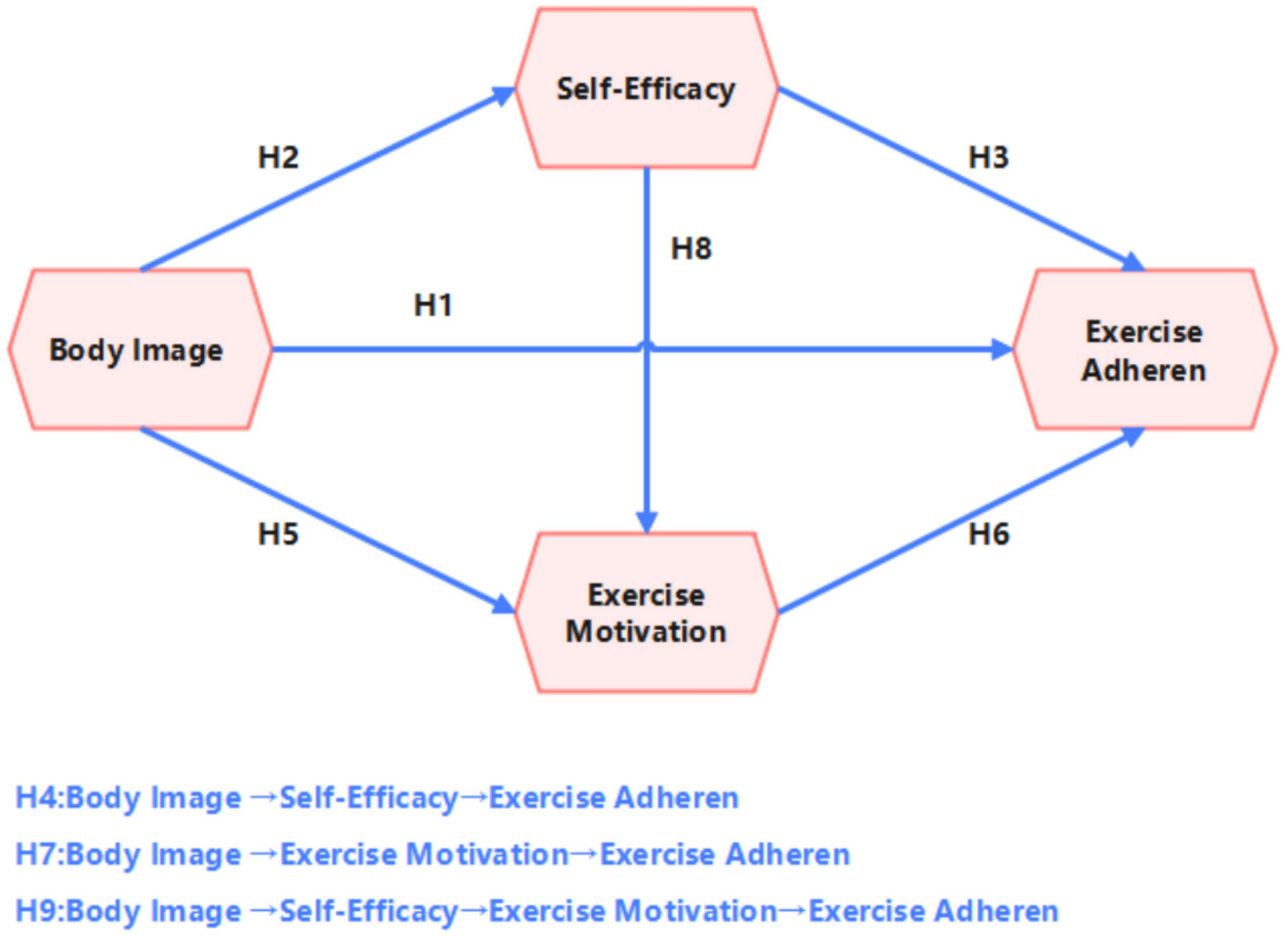
Hypothesized model.

This study employed Model 6 of the PROCESS macro for mediation analysis, a model specifically designed to examine sequential relationships among multiple mediators. This model was selected based on our proposed theoretical framework (see Figure 1), which hypothesizes that body image not only influences fitness persistence behavior through self-efficacy and exercise motivation separately, but also presupposes a progressive psychological pathway of ‘cognitive evaluation → efficacy belief → motivational activation’. PROCESS Model 6 can simultaneously estimate the effect sizes and confidence intervals of these three indirect pathways (i.e., the separate pathway through self-efficacy, the separate pathway through exercise motivation, and the chain pathway through self-efficacy → exercise motivation), thereby precisely deconstructing the underlying psychological mechanisms through which body image influences women’s fitness persistence and achieving a rigorous correspondence between theoretical hypotheses and statistical models.

## 3 Research methods

### 3.1 Survey subjects

The age restriction to women aged 18–38 years was adopted for three reasons. First, this period captures early to middle adulthood, during which concerns about body image tend to be particularly salient and fitness participation is more likely to be shaped by body image–related factors. Second, this age range is commonly used in related research, which facilitates comparability with the existing literature. Third, although the findings should not be generalized directly to females younger than 18 or older than 38, the results remain practically meaningful because women aged 18–38 constitute a primary target group for body image–related fitness interventions.

A total of 750 paper questionnaires were distributed. After excluding invalid responses (e.g., patterned responding or incomplete questionnaires), 721 valid questionnaires were retained. This sample size exceeded the commonly recommended threshold of at least 10 respondents per item (46 items), resulting in an effective response rate of 96.1%. The sample included comparable proportions of married (51.0%) and unmarried (49.0%) participants. Regarding residence, participants were almost evenly distributed between urban (51.2%) and rural (48.8%) areas. The age distribution was relatively even across the four groups: 18–23 years (24.8%), 24–28 years (24.5%), 29–33 years (24.8%), and 34–38 years (25.8%), with a slightly higher proportion in the 34–38 group. In terms of monthly income, 26.1% reported earning 2,000–3,000 RMB and 25.8% reported earning more than 4,000 RMB; those earning below 2,000 RMB and 3,000–4,000 RMB accounted for 23.7 and 24.4%, respectively. Overall, the sample showed a broadly balanced distribution across marital status, residence, age, and income, capturing a wide range of socio-economic characteristics. Detailed information on sample selection and characteristics is presented in Table 1.

**Table 1:** Distribution of demographic characteristics of the study sample (*N* = 721).

Variable	Category	Frequency	Percentage (%)
Marital Status	Married	368	51
	Unmarried	353	49
Residence	Rural	352	48.8
	Urban	369	51.2
Age (years)	18 ~ 23	179	24.8
	24 ~ 28	177	24.5
	29 ~ 33	179	24.8
	34 ~ 38	186	25.8
Monthly income	0 ~ 2000	171	23.7
	2000 ~ 3,000	188	26.1
	3,000 ~ 4,000	176	24.4
	Over 4,000	186	25.8

### 3.2 Research instruments

#### 3.2.1 Body image scale

The Chinese version of the Body Image States Scale (BISS), originally developed by Cash et al. (2002) and translated by Ou Yang (Möri et al., 2022), was used to assess body image. The scale comprises 6 items rated on a 5-point Likert scale (1 = “very dissatisfied” to 5 = “very satisfied”). Higher scores indicate a more positive body image. In this study, the KMO value for the scale was 0.858 and the Bartlett’s test of sphericity was 1071.880 (*p* < 0.001), indicating good construct validity; Cronbach’s *α* was 0.800, demonstrating acceptable reliability. The confirmatory factor analysis results of the scale showed that *χ*^2^/df = 2.282, GFI = 0.989, TLI = 0.974, and RMSEA = 0.042. These findings indicate that the questionnaire has good structural validity.

#### 3.2.2 Exercise persistence scale

This scale was revised by Wang Jiahui based on the “Persistent Scale of Amateur Sports Exercise” compiled by Wang Shen (Wang et al., 2016; Chaudhary and Dhillon, 2022), was utilized. This scale contains 15 items across three dimensions: exercise behavior, effort investment, and emotional experience. Items are scored on a 5-point Likert scale, where 1 indicates “not true” and 5 indicates “completely true.” The total score is calculated by summing the scores of all items, with higher total scores signifying stronger exercise persistence. In this study, the scale yielded a KMO value of 0.945 and a Bartlett’s test of sphericity of 2594.556 (*p* < 0.001), indicating good construct validity; Cronbach’s *α* was 0.869, indicating high reliability. The confirmatory factor analysis results of the scale showed that *χ*^2^/df = 1.189, GFI = 0.993, TLI = 0.991, and RMSEA = 0.016. These findings indicate that the questionnaire has good structural validity.

#### 3.2.3 Self-efficacy

Self-efficacy was measured using the General Self-Efficacy Scale (Luo and Xie, 2023), which consists of 10 items. Each item is rated on a scale from 1 (“completely incorrect”) to 4 (“completely correct”), with total scores ranging from 10 to 40. Higher scores indicate stronger self-efficacy. In this study, the KMO value for the scale was 0.902 and the Bartlett’s test of sphericity was 1709.408 (*p* < 0.001), demonstrating good construct validity; Cronbach’s *α* was 0.836, indicating satisfactory reliability. The confirmatory factor analysis results of the scale showed that *χ*^2^/df = 1.326, GFI = 0.993, TLI = 0.989, and RMSEA = 0.021. These findings indicate that the questionnaire has good structural validity.

#### 3.2.4 Exercise motivation scale

The study employed the simplified version of the Motives for Physical Activities Measure-Revised (MPAM-R) as revised by Shanping et al. (2013). This scale covers five dimensions: appearance motivation, health motivation, enjoyment motivation, competence motivation, and social motivation, with 3 items per dimension. It uses a 5-point Likert scale (1 = “not at all” to 5 = “very strongly”), and the total score, obtained by summing all items, ranges from 15 to 75. Higher scores indicate stronger exercise motivation. In this study, the scale demonstrated a KMO value of 0.946 and a Bartlett’s test of sphericity of 2767.759 (*p* < 0.001), indicating sound construct validity; Cronbach’s *α* was 0.875, confirming good reliability. The confirmatory factor analysis results of the scale showed that *χ*^2^/df = 1.531, GFI = 0.982, TLI = 0.974, and RMSEA = 0.027. These findings indicate that the questionnaire has good structural validity.

### 3.3 Data processing and analysis

Data were analyzed using SPSS 27.0 and PROCESS (version 4.0). Analyses proceeded in five steps. First, data were prepared by coding, screening, and computing composite scores based on valid responses. Second, potential common method bias was assessed using Harman’s single-factor test. Third, reliability and construct validity were evaluated, including internal consistency (Cronbach’s *α*) and suitability for factor analysis (e.g., Bartlett’s test). Fourth, descriptive statistics and bivariate correlations were computed, and linear regression analyses were conducted to examine associations among key variables and to evaluate the effects of body image, self-efficacy, and exercise motivation on women’s fitness persistence. Fifth, mediation effects—including both single-mediator and sequential (chain) mediation—were tested using bootstrap confidence intervals within PROCESS.

This study uses a mediation framework to examine whether self-efficacy and exercise motivation sequentially account for the association between body image and fitness persistence. Importantly, the mediation analysis is based on cross-sectional data and therefore reflects statistical modeling of contemporaneous associations and plausible indirect pathways rather than evidence of causal processes. The ordering of variables is theoretically specified based on prior literature, but it is not empirically established via longitudinal designs or experimental manipulation. Accordingly, the results should be interpreted as patterns of covariation among variables under statistical control. Their value lies in providing preliminary empirical support for the proposed theoretical mechanism and informing future research, rather than supporting direct causal inference.

## 4 Research results

### 4.1 Common method bias test

Statistical methods were used to test for common method bias. Harman’s single-factor test was performed by conducting an exploratory factor analysis (EFA) on all measurement items. The results showed that the variance explained by the first unrotated factor was 30.162%, which is below the critical threshold of 40%, indicating that there is no serious common method bias in the data. Although a single factor did not account for the majority of the variance, additional measures were implemented in the questionnaire design to control for potential bias:

1) Employing anonymous responses and including reverse-scored items.
2) Separating the order of items for independent and dependent variables.
3) Using multi-source scales with clearly stated items. Subsequent analyses could also incorporate methods such as controlling for unmeasured latent method factors or introducing marker variables for supplementary verification. In summary, the data quality of this study meets the basic requirements for statistical analysis.

As shown in Table 2, the study examined the constructed theoretical framework through structural equation modeling in terms of model fit evaluation, with the actual measured values of each fit indicator presented in Table 2. Specifically, the chi-square degrees of freedom ratio (*χ*^2^/df) was 1.092, which is below the recommended threshold of 3, indicating a good model fit. The root mean square error of approximation (RMSEA) was 0.011, far below the stringent criterion of 0.05, suggesting minimal model error. The comparative fit index (CFI) was 0.991, the incremental fit index (IFI) was 0.991, and the Tucker–Lewis index (TLI) was 0.990, all exceeding the ideal level of 0.95, further confirming a high degree of alignment between the model and the data. In summary, all fit indicators met or surpassed widely recognized academic standards, indicating that the chain mediation model constructed in this study demonstrates good overall goodness of fit and effectively reflects the structural relationships among the variables.

**Table 2:** Actual measured values of model fitting degree evaluation indicators.

*χ*^2^/df	RMSEA	CFI	IFI	TLI
1.092	0.011	0.991	0.991	0.990

### 4.2 Correlation analysis of research variables

Descriptive statistics (means and standard deviations) and bivariate correlations were computed for all study variables (Table 3). The mean scores for body image (*M* = 3.57, SD = 0.78), fitness persistence (*M* = 3.57, SD = 0.58), self-efficacy (*M* = 3.25, SD = 0.71), and exercise motivation (*M* = 3.39, SD = 0.66) were all above the theoretical midpoint, indicating generally favorable levels of body-related perceptions and exercise-related tendencies in this sample. Correlation analyses showed that all variables were significantly and positively related (*p*s < 0.01). The strongest association was observed between body image and self-efficacy (*r* = 0.829), followed by the association between self-efficacy and exercise motivation (*r* = 0.713). Body image was also moderately to strongly associated with fitness persistence (*r* = 0.695), and exercise motivation was similarly related to fitness persistence (*r* = 0.672).

**Table 3:** Mean, standard deviation and correlation coefficient of each research variable.

	*M*	SD	BI	EA	SE	EM
BI	3.57	0.78				
EA	3.57	0.58	0.695^***^			
SE	3.25	0.71	0.829^***^	0.684^***^		
EM	3.39	0.66	0.672^***^	0.672^***^	0.713^***^	

^*^ indicates a significance level of*p* < 0.05, ^**^ indicates a significance level of *p* < 0.01, ^***^ indicates a significance level of *p* < 0.001; Body Image for short BI, Exercise Adherence for short EA, Self-Efficacy for short SE, Exercise Motivation for short EM, the same below. Multiple Linear Regression Analysis.

Overall, these correlational patterns are consistent with the proposed framework in which more positive body image is associated with higher self-efficacy, which is in turn related to greater exercise motivation and stronger fitness persistence. Although several correlations were relatively high, the common method bias assessment suggested that the results were unlikely to be driven primarily by a single-method factor (the first factor accounted for 30.16% of the variance), providing preliminary support for the robustness of the observed associations.

### 4.3 Multiple linear regression analysis

Table 4 presents the results of the multicollinearity diagnosis among the independent variables in this study. Tolerance and the Variance Inflation Factor (VIF) were used as evaluation criteria. Generally, if the tolerance is below 0.10 or the VIF exceeds 10, it indicates a serious multicollinearity problem.

**Table 4:** Multicollinearity diagnosis.

Variable	Tolerance	VIF
BI	0.299	3.345
SE	0.267	3.379
EM	0.470	2.126

As shown in Table 4, the tolerance values for the three independent variables are as follows: Body Image (BI) is 0.299, Self-Efficacy (SE) is 0.267, and Exercise Motivation (EM) is 0.470. The corresponding VIF values are 3.345, 3.379, and 2.126, respectively. All variables have tolerance values above 0.10 and VIF values below 10, indicating that there is no serious multicollinearity issue among the variables, which meets the requirements for further regression analysis.

(1) The independent predictive effect analysis for women’s fitness adherence behavior (see Table 5) indicates that body image, self-efficacy, and exercise motivation all have significant positive explanatory power. Specifically:

**Table 5:** Individual regression analysis of body image, self-efficacy and exercise motivation on women’s exercise adherence.

Variable	Female exercise adherence						
	*B*	SE	*β*	*T*	*F*	*R* ^2^	*R* ^2^_adj_
Constant	1.724	0.073					
BI	0.516	0.020	0.695	25.945	673.147	0.189	0.187
Constant	1.753	0.074					
SE	0.558	0.022	0.684	25.173	633.656	0.468	0.467
Constant	1.548	0.084					
EM	0.596	0.025	0.672	24.332	592.052	0.452	0.451

*R*^2^_adj_ indicates adjusted *R*^2^.

(2) Body image has the highest standardized regression coefficient (*β* = 0.695, *p* < 0.001) and independently accounts for 18.7% of the variance (*R*^2^_adj_ = 0.187), suggesting that a positive evaluation of one’s own body is a key driving factor for fitness behavior.

(3) Self-efficacy exhibits the strongest explanatory power (*β* = 0.684, *p* < 0.001; *R*^2^adj = 0.467), implying that increased confidence in achieving fitness goals can enhance behavioral adherence by nearly 46.7%.

Although the predictive effect of exercise motivation is slightly lower, it remains significant (*β* = 0.672, *p* < 0.001; *R*^2^adj = 0.451), reflecting the critical role of intrinsic drive in sustaining behavior.

All models reached highly significant *F*-values (*F* > 592.052), and the standardized coefficients all exceed 0.65, meeting the criteria for strong effects. These findings support hypotheses H1, H3, and H5.

### 4.4 Mediation effect test

A bias-corrected bootstrap procedure with 5,000 resamples was conducted using PROCESS Model 6 to test the sequential (chain) mediation model. Marital status, residence, age, and monthly income were included as covariates to account for potential confounding. As shown in Table 6, body image demonstrated a significant total effect on women’s fitness persistence (effect = 0.515, SE = 0.020, 95% CI [0.476, 0.554]). When mediators were included, the direct effect of body image remained significant (effect = 0.236, 95% CI [0.170, 0.303]), representing 45.9% of the total effect. The total indirect effect was also significant (effect = 0.279, 95% CI [0.230, 0.332]), accounting for the remaining 54.1%, indicating that self-efficacy and exercise motivation jointly explained a substantial portion of the association between body image and fitness persistence.

**Table 6:** Test results of standardized paths for mediating effects.

Variable	Path	Effect size	Standard error	LLCL	ULCL	Effect ratio
Total effect	Direct path	0.5150	0.0200	0.4758	0.5543	100%
Direct effect		0.2363	0.0338	0.1699	0.3026	45.9%
Total indirect effect		0.2787	0.027	0.23	0.3317	54.1%
Indirect effects	Path 1	0.1173	0.0272	0.06	0.1703	22.8%
	Path 2	0.0612	0.0116	0.0397	0.0855	11.9%
	Path 3	0.1002	0.0125	0.0766	0.1258	19.4%

We employed regression-based mediation analysis in PROCESS rather than full structural equation modeling (SEM) primarily for analytical parsimony and alignment with the study design. Specifically, the mediation tests were conducted on observed composite scores, and the PROCESS framework provides a straightforward approach for estimating indirect effects with bootstrap confidence intervals while controlling for covariates. Although SEM offers advantages—particularly when modeling latent variables and simultaneously accounting for measurement error—it typically involves greater model complexity and additional parameter estimation, which can increase demands on sample size and model identification. Accordingly, our regression-based approach was selected to reduce unnecessary model complexity and to provide stable estimates of indirect effects within the present analytic framework. Nevertheless, the sample size and sampling scope may limit generalizability, and future studies could use larger and more diverse samples—and, where appropriate, latent-variable SEM with longitudinal designs—to further validate the proposed mechanism.

**Figure 2:**
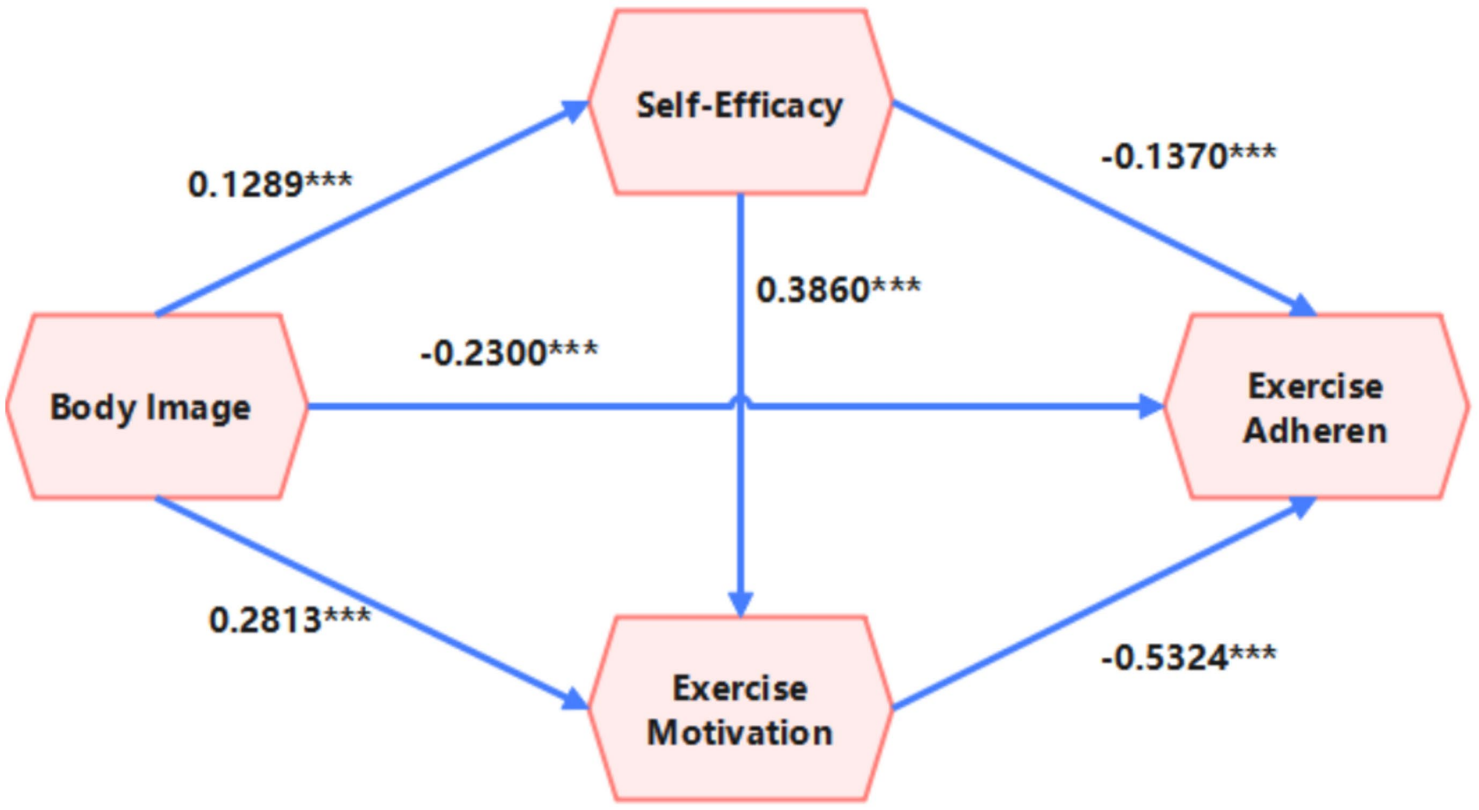
Chain mediation model diagram of self-efficacy and exercise motivation.

Decomposition of mediation pathways is as follows (see Figure 2):

Pathway 1 (Body Image → Self-Efficacy → Fitness Behavior): The independent mediation effect is 0.117 (accounting for 22.8, 95% CI [0.060, 0.170]). This is the strongest mediation it consconsently that among the three pathways, aligning with the core principle of social cognitive theory, which posits that cognitive evaluation enhances efficacy beliefs, directly driving behavior.

Pathway 2 (Body Image → Exercise Motivation → Fitness Behavior): The independent mediation effect is 0.061 (accounting for 11.9, 95% CI [0.040, 0.086]). Although its effect size is lower than the other pathways, the confidence interval does not include zero, indicating that the direct impact of body image on exercise motivation remains statistically significant.

Pathway 3 (Body Image → Self-Efficacy → Exercise Motivation → Fitness Behavior): The chained mediation effect is 0.100 (accounting for 19.4, 95% CI [0.077, 0.126]). This suggests that self-efficacy not only directly influences behavior but also enhances behavioral persistence by strengthening exercise motivation. This supports the multi-level integrated model of “Cognition → Belief → Motivation.”

Pathway strength ranking: Pathway 1 (single-step mediation) > Pathway 3 (chained mediation) > Pathway 2 (single-step mediation). This indicates that self-efficacy has the strongest explanatory power in the relationship between body image and fitness behavior, whereas the independent mediation effect of motivation is relatively limited.

Model Integrity Validation: The sum of the effect values of the three pathways (0.1173 + 0.1002 + 0.0612 = 0.2787) perfectly matches the total mediation effect (0.2787), confirming that the model does not omit any significant mediation pathways.

## 5 Discussion

Grounded in Social Cognitive Theory and Self-Determination Theory, this study constructed and empirically tested a chain mediation model to explain women’s fitness persistence behavior. Path analysis revealed that the positive effect of body image on fitness persistence is primarily achieved through the individual and sequential mediating roles of self-efficacy and exercise motivation, thereby clarifying the validity of the “cognition-belief-motivation” psychological transmission pathway. Notably, the observed strong positive correlations between body image and self-efficacy, as well as between self-efficacy and exercise motivation, warrant careful interpretation within this integrated theoretical framework. Rather than indicating statistical redundancy, these strong associations reflect the close coupling and fluid transformation of cognitive evaluations into efficacy beliefs and subsequent motivational regulation within the fitness context. When women hold positive evaluations of their body image, their confidence in successfully executing fitness behaviors is correspondingly strengthened, and this efficacy belief further facilitates the activation of intrinsic motivation and autonomous regulation. Among these pathways, self-efficacy emerged as the most potent mediator, underscoring its role as the core engine linking body-related cognition to behavioral persistence. Furthermore, the present sample—comprising physically active women in a life stage characterized by heightened body awareness—may exhibit particularly pronounced synergistic enhancement between body image and psychological resources. This effect may be further amplified by the contemporary Chinese sociocultural context, in which traditional “slimness culture” intertwines with the emerging discourse of “healthy beauty,” rendering women’s evaluations of their bodies closely linked not only to external aesthetics but also to social identity and self-worth. In sum, these findings not only elucidate the complex interrelationships among body image, self-efficacy, and exercise motivation, but also provide robust empirical support for an integrated theoretical model bridging cognitive evaluation and motivational regulation. Importantly, they identify specific psychological leverage points—namely, body image reconstruction and efficacy belief strengthening—for designing targeted interventions aimed at reshaping body-related cognition, consolidating efficacy beliefs, and thereby stimulating autonomous exercise motivation in female populations.

### 5.1 Impact of body image on women’s fitness adherence behavior

This study identifies a positive association between body image and women’s fitness behavior, underscoring the interplay between psychological self-construction and behavioral choices within contemporary sociocultural environments. From a motivational formation perspective, when women develop a more positive body image—characterized by greater autonomy and a stronger sense of competence—fitness participation may shift from an externally driven obligation to an expression of self-identity. Women with higher body appreciation, for example, are more likely to frame exercise as self-care rather than as aesthetic discipline (Jin et al., 2015).

Notably, the emergence of more internalized motivation may coincide with changes in cognitive resource allocation. Negative body image can elicit rumination that consumes executive resources, whereas a more positive body image may facilitate a more adaptive mind–body state—potentially through stress-related physiological pathways—thereby supporting sustained exercise engagement (Harwood and Knight, 2009). Sociocultural influences also appear to shape how motivational processes operate. Body-related content on social media can create behavioral reference points through social comparison, yet its effects may depend on perceived attainability. Specifically, upward comparisons may be more likely to translate into sustained motivation only when individuals believe that their ideal physique is achievable through effort (Lee et al., 2019).

These findings also invite a reconsideration of conventional fitness-promotion strategies. Approaches that emphasize external appearance-based incentives may produce short-term behavioral change but can inadvertently intensify self-objectification and cognitive strain, thereby undermining long-term persistence. In contrast, interventions that support body autonomy and embodied agency—such as embodied movement practices (e.g., dance-based programs)—may be better positioned to foster sustainable engagement. In this sense, fitness persistence can be understood as a process through which women renegotiate embodied subjectivity; lasting exercise autonomy is more likely when women are able to move beyond being positioned primarily as “bodies to be observed.”

### 5.2 The mediating role of self-efficacy

According to Bandura’s social cognitive theory, self-efficacy refers to individuals’ beliefs in their capability to organize and execute actions required to attain specific goals, and it directly is related to goal setting, effort investment, and persistence in the face of obstacles (Bandura, 1977). In the fitness context, women’s perceptions of their body image may influence efficacy beliefs such as “I can persist in exercising,” thereby relating to longer-term adherence to fitness behavior (Law and Hall, 2009).

A more positive body image is associated with confidence in exercise capability. When women observe improvements in physical functioning through regular training, their perceived efficacy for completing more demanding exercise may increase, which can support continued engagement (Tylka and Wood-Barcalow, 2015). In contrast, negative body image may undermine self-efficacy and foster maladaptive appraisals—such as perceiving effort as ineffective—thereby increasing the likelihood of discontinuation (Larson et al., 2019).

Self-efficacy may support adherence by buffering negative affective responses to setbacks. Women with higher self-efficacy tend to interpret exercise-related difficulties as temporary and manageable rather than as evidence of personal inadequacy, and this form of cognitive reappraisal can reduce the disruptive impact of anxiety and frustration on behavior (Michie et al., 2013). For example, Annesi and Powell’s (2024) longitudinal study reported that during plateau periods, women with higher self-efficacy showed greater emotional stability and lower dropout risk than those with lower self-efficacy (Slater and Tiggemann, 2010).

### 5.3 The mediating role of exercise motivation

According to self-determination theory, intrinsic motivation and more internalized forms of regulation (e.g., integrated regulation) are conducive to sustained behavioral commitment. When women hold a more positive body image, they may be more likely to construe fitness as a pathway to self-realization, thereby fostering autonomous motivation. In contrast, exercise goals primarily driven by appearance-related anxiety tend to elicit controlled motivation, which is often associated with declines in adherence once external pressures or contingencies are removed (Standage and Gillison, 2007).

Body image may be indirectly associated with behavior indirectly by shaping individuals’ appraisals of their exercise capabilities. A more positive body image can strengthen women’s confidence in their physical ability, which may increase willingness to persist. Motivational quality further differentiates goal setting: individuals with autonomous motivation more often adopt mastery-oriented goals, whereas those with controlled motivation are more likely to pursue performance-oriented goals. Longitudinal evidence suggests that mastery goals enhance enjoyment during exercise, which in turn supports adherence over time (Dunn et al., 2016).

In addition, sociocultural exposure to “ideal body” imagery on social media can intensify appearance-focused attention and shift exercise motives from health management toward appearance-based competition (Tiggemann et al., 2018). Under such conditions, even initially autonomous motives may become increasingly contingent on external standards, potentially undermining sustained engagement.

### 5.4 The chain mediation role of self-efficacy and exercise motivation

Self-efficacy represents an important motivational resource for exercise. Individuals with higher self-efficacy are more likely to set challenging goals, sustain effort, and maintain motivational engagement even when encountering setbacks. In the fitness context, when women feel confident in their exercise capabilities, they are more likely to endorse stronger and more internalized reasons for participation. By supporting the satisfaction of the need for competence, self-efficacy can facilitate autonomous motivation (e.g., intrinsic interest and personally endorsed goals) and thereby promote continued exercise participation.

Body image may be dynamically shaped and translated into behavior through psychological resources. A more positive evaluation of one’s body can strengthen confidence in exercise capability and subsequently be expressed as clearer and more self-determined exercise motivation. This proposed sequential pathway reflects a multi-stage psychological process that evolves from cognitive appraisal to motivational activation. Supporting this account, Jankauskiene and Baceviciene (2022) reported that the indirect pathway from body image to exercise behavior through self-efficacy accounted for 68% of the total effect (Essiet et al., 2021).

In addition, positive body image—characterized by lower self-criticism and greater body acceptance—may reinforce confidence in one’s capacity to exercise. For example, when women perceive their appearance as closer to prevailing aesthetic ideals, they may be more willing to try new exercise programs (Themanson et al., 2019). Related evidence from neuroimaging research has also suggested that body satisfaction may be linked to activity in prefrontal regions implicated in self-regulation and goal persistence (please ensure an appropriate citation is provided if this point is retained).

## 6 Conclusion and prospect

### 6.1 Conclusion

Grounded in Social Cognitive Theory and a multi-level integrative framework, this study examines the mechanisms underlying women’s fitness persistence by clarifying the interrelations among body image, self-efficacy, and exercise motivation. The findings indicate that more positive body-related cognitions are associated with stronger fitness persistence not only directly but also indirectly through enhanced self-efficacy and more autonomous forms of exercise motivation. Self-efficacy appears to play a central role: it functions as an independent mediator linking body image with fitness persistence and also operates within a sequential pathway in which self-efficacy is associated with exercise motivation, jointly accounting for additional variance in persistence. Overall, the results provide empirical support for a “cognition–belief–motivation” integrative framework in understanding fitness behavior and underscore the joint contributions of cognitive appraisals and motivational processes to sustained engagement.

### 6.2 Research limitations

Although this study provides empirical evidence regarding the association between body image and women’s fitness persistence and the proposed sequential mediation pathways, several limitations should be acknowledged.

First, the sample was restricted to women aged 18–38 years in Shandong Province. While the sample was relatively balanced across marital status, residence, age, and income, China is characterized by substantial regional and cultural heterogeneity. Women’s exposure to—and internalization of—discourses such as “thinness culture” and “healthy beauty” may vary across regions, and the generalizability of the present findings therefore warrants further examination in more diverse geographic and cultural settings.

Second, the cross-sectional design captures contemporaneous associations but cannot track temporal changes in body image, self-efficacy, and exercise motivation. As a result, the proposed mediation pathways should be interpreted as plausible indirect associations rather than evidence of causal sequencing. Future research using longitudinal follow-up designs, experience-sampling approaches, or cross-lagged panel models would help clarify directionality and dynamic change.

Third, demographic factors were not included as covariates in the regression and mediation models. Although preliminary analyses suggested that these variables were not significantly associated with the focal constructs, future studies could incorporate demographic characteristics as controls or examine their potential moderating roles using larger and more heterogeneous samples to improve explanatory precision and external validity.

In addition, reliance on self-reported measures may introduce social desirability bias and recall error. For example, societal appearance norms could lead some participants to underreport negative body-related experiences or overreport persistence, contributing to discrepancies between reported and actual behavior. Incorporating objective indicators (e.g., attendance records, wearable-based activity indices) or multi-informant data could strengthen measurement validity.

Moreover, although the bootstrap-based mediation analysis supported indirect effects, a substantial portion of the total effect remained unexplained (45.9% of the total effect). This suggests that other mediators or moderators—such as social support, economic constraints, access to facilities, or health status—may also contribute to fitness persistence and should be examined in future work. Relatedly, the sequential mediation model provides statistical evidence consistent with the proposed mechanism, but it remains correlational and does not permit strong causal conclusions.

Finally, while this study acknowledged that cultural values may shape motivational processes, it did not directly test how tensions between emerging “functional beauty” discourses and appearance-centered standards in China relate to women’s fitness psychology. Given the complexity and fluidity of cultural contexts, future research would benefit from more nuanced theorizing and empirical strategies, including longitudinal tracking, multi-source data, and cross-cultural comparisons, to further refine and validate the proposed framework.

### 6.3 Future outlook

To address the limitations of cross-sectional evidence, future research should establish long-term tracking cohorts and adopt mixed-methods designs to capture dynamic, reciprocal changes among body image, self-efficacy, and exercise motivation. Building on the mediation pathways identified in the present study and prior literature, differentiated and population-specific strategies could be developed for diverse groups. In particular, an enhanced social–ecological framework may help articulate a “resources–psychology–behavior” coupling model of fitness participation, enabling quantitative tests of how environmental inputs—such as accessibility of public fitness facilities and the professionalism of coaches—influence self-efficacy and subsequent persistence.

Methodologically, spatial analysis using Geographic Information Systems (GIS) could be leveraged to identify mismatches between resource allocation and areas with low exercise persistence, and to inform facility placement and program design aligned with group-level psychological characteristics. At the intervention level, future studies should translate psychological evidence into modules that are measurable, actionable, and scalable—for example, standardized components targeting body image, efficacy beliefs, and motivational regulation that can be implemented across settings and evaluated with common outcome metrics. By integrating longitudinal mechanism testing, stratified intervention design, and attention to diverse populations, future work may contribute to a closed-loop system that links individual-level cognitive and motivational change with broader social and environmental support.

## Data Availability

The original contributions presented in the study are included in the article/Supplementary material, further inquiries can be directed to the corresponding author.
